# Different MMSE Score Is Associated with Postoperative Delirium in Young-Old and Old-Old Adults

**DOI:** 10.1371/journal.pone.0139879

**Published:** 2015-10-13

**Authors:** Yujie Wu, Zhongyong Shi, Meijuan Wang, Yingbo Zhu, Cheng Li, Guodong Li, Edward R. Marcantonio, Zhongcong Xie, Yuan Shen

**Affiliations:** 1 Department of Psychiatry, Tenth People’s Hospital of Tongji University, Shanghai 200072, P. R. China; 2 Department of Anesthesiology, Tenth People’s Hospital of Tongji University, Shanghai 200072, P. R. China; 3 Department of Orthopedic Surgery, Tenth People’s Hospital of Tongji University, Shanghai 200072, P. R. China; 4 Divisions of General Medicine and Primary Care and Gerontology, Department of Medicine, Beth Israel Deaconess Medical Center and Harvard Medical School, Boston, Massachusetts 02215, United States of America; 5 Geriatric Anesthesia Research Unit, Department of Anesthesia, Critical Care and Pain Medicine, Massachusetts General Hospital and Harvard Medical School, Charlestown, Massachusetts 02129–2060, United States of America; University of Pennsylvania, UNITED STATES

## Abstract

**Background:**

Postoperative delirium is one of the most common postoperative complications in geriatric patients. Mini-mental state examination (MMSE) assesses cognitive function in patients and is associated with postoperative delirium. However, whether there is an age-dependent relationship between preoperative MMSE score and postoperative delirium remains unknown.

**Methods:**

We therefore set out to investigate the association between preoperative MMSE score and postoperative delirium in young-old (≤80 year-old, 75.46±4.69 years, 27.0% male, n = 63) and old-old (>80 year-old, 84.51±3.46 years, 20.9% male, n = 67) participants, who had repairs of hip fractures under general anesthesia. The Confusion Assessment Method and Memorial Delirium Assessment Scale were administrated before surgery, and on the first, second and fourth days after surgery, to assess the incidence and severity of the delirium, respectively. A receiver operating characteristic curve analysis was used to calculate the optimal cutoff score of MMSE in predicting postoperative delirium.

**Results:**

Thirty-four (26.2%) of 130 patients (80.12±6.12 years, 23.8% male) developed postoperative delirium. Preoperative MMSE scores were negatively associated with higher incidences and greater severity of postoperative delirium. The optimal cutoff scores of MMSE associated with postoperative delirium for young-old and old-old participants were 18.4 and 21.4, with a sensitivity of 60% and 83.8%, and a specificity of 92.5% and 62.8%, respectively.

**Conclusion:**

The data demonstrated the optimal cutoff score of MMSE associated with postoperative delirium in young-old adults might be lower than that in old-old adults. Pending further investigation, these findings suggest that the association between preoperative MMSE score and postoperative delirium is age-dependent.

## Introduction

Postoperative delirium is one of the most common complications in elderly patients [1,2], and has independent, adverse effects on short and long-term mortality and morbidity, including poor functional recovery, postoperative cognitive dysfunction, deterioration in quality of life, and increased costs of medical care ([3–6], reviewed in [7,8]). Mini-mental state examination (MMSE) measures cognitive function, and preoperative cognitive function is associated with postoperative delirium [1]. Moreover, MMSE has been reported to be associated with postoperative delirium [4]. However, it is unknown whether the optimal cutoff score of MMSE associated with postoperative delirium are different in patients with different age, e.g., young-old (≤ 80 year-old) versus old-old (> 80 year-old) adults.

We therefore conducted this prospective study using a cohort of elderly patients, who had hip repair surgeries for hip fractures under general anesthesia, to determine whether different preoperative MMSE scores were associated with postoperative delirium in young-old and old-old participants, respectively. The objective of the study was to determine an age-dependent optimal preoperative MMSE cutoff score that is associated with postoperative delirium in two groups of patients: younger than 80 years old (young-old) and older than 80 years old (old-old). The hypothesis was that the optimal cutoff score of MMSE associated with postoperative delirium in young-old participants would be lower than that in old-old participants. This hypothesis is supported by the literatures that aging and impaired cognitive function are the risk factors of the development of postoperative delirium [1,9–15]. The outcomes from the studies would suggest that old-old patients might still develop postoperative delirium even with less impairment of preoperative cognitive function (e.g., better MMSE score) as compared to young-old patients. We chose the patients who had hip repair surgeries for the current studies because these patients are generally older in age and also have higher risks of developing postoperative delirium. These findings may promote more research to identify the age-dependent association between preoperative cognitive function and postoperative delirium, which would ultimately lead to better understanding the role of age and preoperative cognitive impairment in the development of postoperative delirium.

## Methods

### Participants

**Fig 1:**
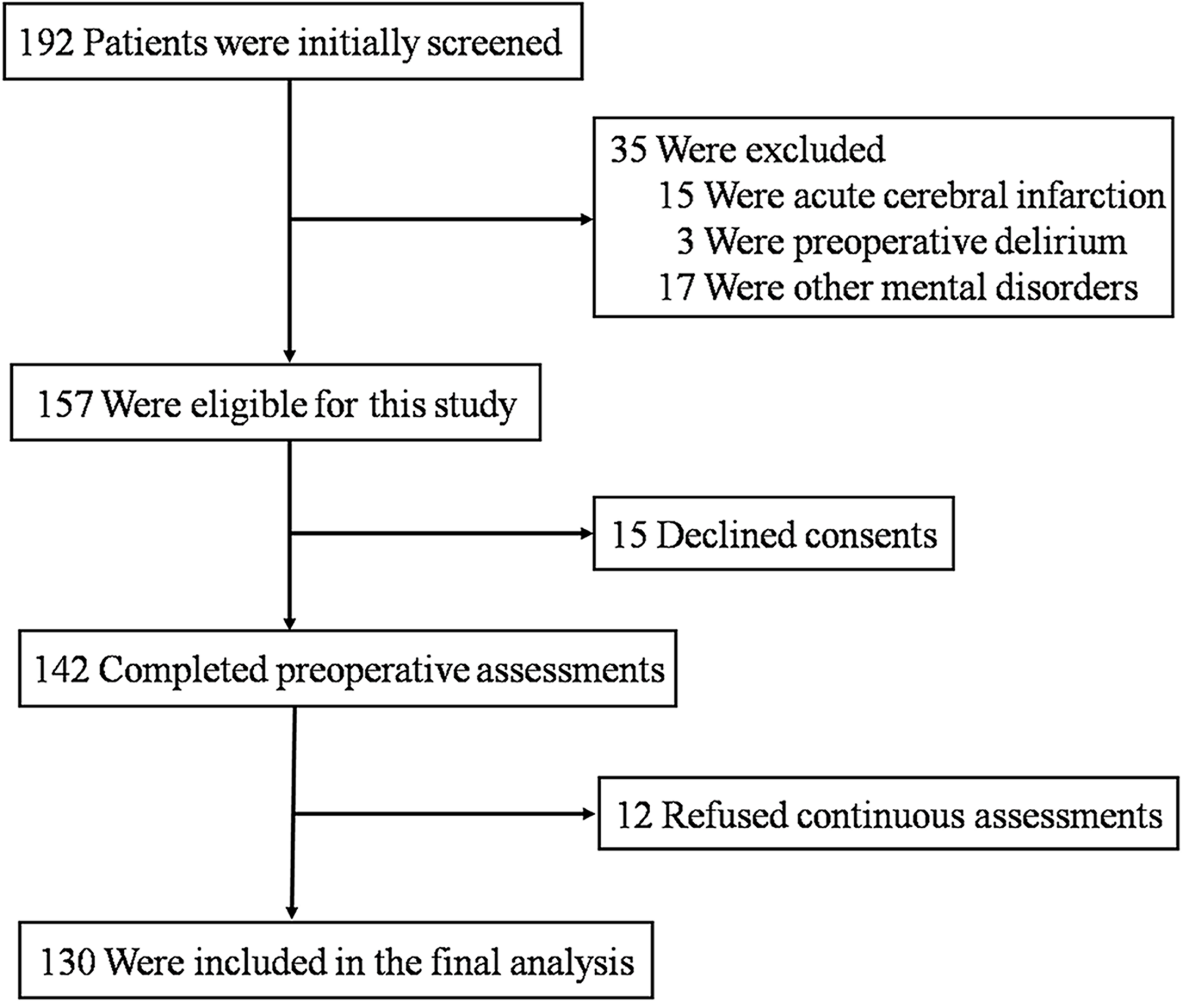
Flow diagram. The flow diagram shows that 192 participants were initially screened for the studies and finally 130 participants were included in the data analysis.

The protocol was approved by the Institutional Ethics Committee of Human Research of the Tenth People's Hospital, Shanghai, China [RES-2013015]. A total of 192 patients, who had hip fractures and were admitted to the Department of Orthopedics in the Tenth People’s Hospital, were screened and asked to participate in the study. Participants were included if they: 1) were 65 years old or older; 2) had hip replacement or open reduction with internal fixation surgery under general anesthesia for the repair of hip fractures. Patients were excluded from participation if they had pre-existing delirium, acute cerebrovascular disorders, or mental disorders (e.g., depression or schizophrenia) diagnosed by using the Diagnostic and Statistical Manual of Mental Disorders (DSM-IV) [16]. We exclude patients with acute cerebrovascular disorders because these patients may have difficulty to perform the cognitive tests and delirium assessment. All participants signed the written informed consent prior to being enrolled in the study. The participants were screened for the study between August, 2013 and August, 2014. One hundred and ninety-two participants were enrolled and the data from 130 participants were included for the final data analysis (see Fig 1, the flow diagram). Sample size was calculated by taking the difference in MMSE scores from the participants with delirium and the participants without delirium in our pilot study, assuming 80% power and 5% type I error.

### Preoperative interview

Screening assessments were performed one day before the scheduled surgery, which was generally two days after the hip fracture. The assessment included demographic characteristics (e.g., age, gender and education), medical information (e.g., diagnosis and type of surgery), and the evaluation of cognitive function using MMSE performed before the treatment of atropine and solumedrol. The preoperative Confusion Assessment Method (CAM) was also performed in the participants the day before their surgeries.

### Anesthesia and surgery

The participants had hip replacement or open reduction with internal fixation surgery under standardized perioperative care. The participants were given midazolam (1.17 ± 0.88 mg, intravenous administration) as a preoperative medication. The general anesthesia was induced by intravenous administration of propofol (54.43 ± 54.97 mg), sufentanil (22.04 ± 9.23μg), and cisatracurium (6.82 ± 6.58 mg). The general anesthesia was maintained by using propofol (299.45 ± 252.98 mg), remifentanil (0.48 ± 0.49 mg), sevoflurane (21.90 ± 10.66 mL), and cisatracurium (8.68 ± 12.97 mg). Many hospitals use regional anesthesia for the hip surgery, but our hospital use general anesthesia for the surgery, which provide us an opportunity to evaluate the postoperative delirium after the hip surgery under general anesthesia. The postoperative pain control included a standard postoperative pain management, e.g., sufentanil and butorphanol patient controlled analgesia (0.5 μg sufentanil and 0.0125 mg butorphanol per injection, interval time of injection was 15 minutes with a total of 2 μg sufentanil and 0.05 mg butorphanol per hour). There were no major complications among the participants during the immediate postoperative period.

### Postoperative interview

The assessment of delirium was performed as described in our previous studies [17]. Specifically, the psychiatrists (Y.W. or Z.S.) interviewed the patients once daily between 3:00 pm and 6:00 pm on the first, second and fourth days after the surgery. Patient charts were not reviewed for episodes of delirium which could have occurred outside of this defined time of assessment. The occurrence of postoperative delirium was assessed by using the CAM diagnostic algorithm and the severity of the postoperative delirium was assessed by using the Memorial Delirium Assessment Scale (MDAS) [18,19].

### Statistics

The One-Sample Kolmogorov-Smirnov method was used first to test the normality of all of the variables. Student *t*-test was used to compare the continuous variables, including demographic (e.g., age, height, weight, education) and clinical characteristics (e.g., length of anesthesia, length of operation, estimation of blood loss), between the participants with postoperative delirium and the participants without postoperative delirium. Mann-Whithney *U* test was used to analyze the MMSE, MDAS and ADL scores between the participants with postoperative delirium and the participants without postoperative delirium. *Chi*-square tests were used to compare the dichotomous factors, e.g., gender.

**Fig 2:**
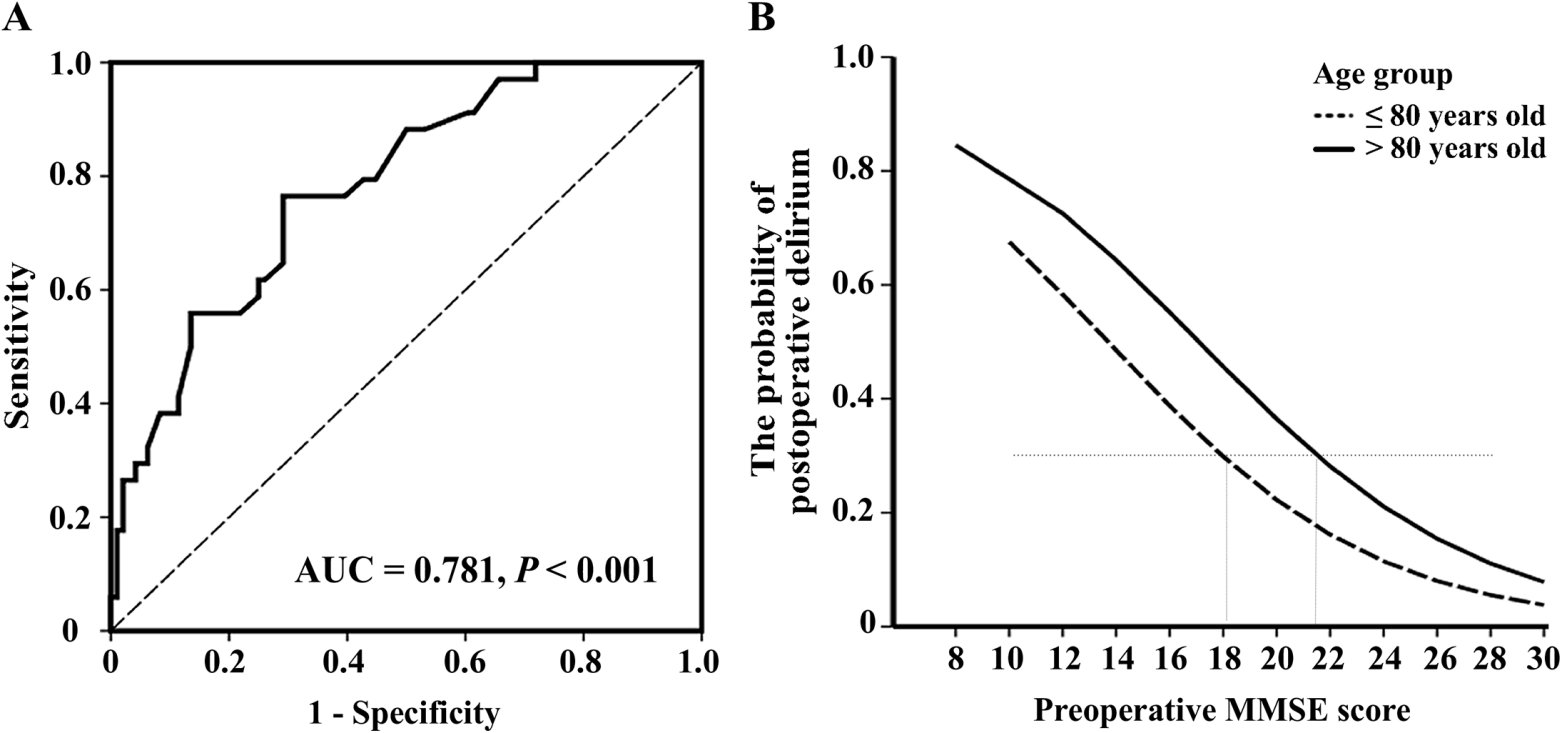
The preoperative MMSE score predicts the postoperative delirium. (A) ROC analysis was used for the determination of the diagnostic sensitivity and the specificity of the preoperative optimum cutoff score of MMSE versus CAM. (B) The relationship of probability of postoperative delirium and the preoperative MMSE score was determined by these formulas: [Logit (*p*) = 2.714–0.198*MMSE] and [Logit (*p*) = 3.263–0.191*MMSE] for the young-old and old-old participants, respectively. The probability of the postoperative delirium decreases with the increase of the MMSE scores, with a linear association in both groups of participants, young-old (dotted line) and old-old (solid line).

Univariate and multivariate logistic regressions were performed to identify the potential risk factors of postoperative delirium. Each variable was screened using the univariate regression and the variables with *P* < 0.25 were selected for multivariate logistic regression. Those variables with *P* < 0.05 in the multivariate regression were defined as having an association with the postoperative delirium [20]. The odd ratio (OR) and 95% confidence interval (95% CI) were used to illustrate the predictive power of certain characters. Moreover, the probability of postoperative delirium (*p*), predicted by preoperative risk factors, was quantified in different age groups. Logit (*p*) = *β*_*0*_ + *β*_*1*_X_1_ + *β*_*2*_X_2_ +……+ *β*_*n*_X_n_ was used to determine the position and the shape of the lines in Fig 2B, in which “X” is the risk factor, “n” is the number of influence factors, *β* is the influence coefficient (indicating how heavily the corresponding independent variables contribute to the outcome), and *β*_*0*_ is a constant generated by SPSS automatically [21]. A Spearman correlation was used to determine the association between preoperative characteristics and postoperative MDAS scores (the mean score at postoperative days 1, 2 and 4). The partial coefficient (adjusted for age, gender and education) was also used to illustrate the association between the preoperative ADL and MMSE scores and the postoperative MDAS score. A receiver operating characteristic (ROC) curve was used to determine the optimal MMSE cutoff score for the diagnosis of CAM-defined postoperative delirium. The total area under the curve (AUC), its 95% confidence interval (CI), the sensitivity, and the specificity, were all used for this determination. The optimal MMSE score was calculated according to Youden index (maximum of [sensitivity + specificity—1]) [22]. All analyses were performed using SPSS version 20.0 (SPSS Inc., Chicago, IL) with *P* < 0.05 as the significance level.

## Results

### Characteristics of participants

One hundred and ninety two patients were screened, 35 of them were not eligible for this study, owing to acute cerebral infarction (n = 15, occurred within three months), preoperative delirium (n = 3), and other mental disorders (n = 17). Fifteen patients declined consent, resulting in an initial study cohort of 142 patients for the preoperative assessments. Among these 142 patients, twelve patients refused to complete the continuous assessments after the surgery. Thus, there were 130 patients who completed both pre- and post-operative assessments, and were entered into the final analysis of the data (see Fig 1, the flow diagram). The demographic and clinical data of the participants is presented in Table 1. All of the participants had either hip replacement surgeries (N = 68) or open reduction and internal fixation surgeries (ORIF, N = 62) under general anesthesia. Thirty-four of the 130 participants (26.2%) developed postoperative delirium on postoperative days 1, 2, or 4, including hyperactive delirium (15/34, 44.1%), hypoactive delirium (13/34, 38.2%), as well as mixed delirium with both hyperactive and hypoactive features (6/34, 17.7%). The mean MMSE score of the participants was 22.23 ± 5.44, among which 52 (52/130, 40.0%) participants had MMSE ≥ 24 and 53 (53/130, 40.8%) had MMSE score between 18 and 24. Other 25 (25/130, 19.2%) participants had MMSE score ≤ 17. Note MMSE 17 is the defined cutoff score as one of the diagnostic criteria for dementia. MMSE 18 to 24 is the defined cutoff score, which can help to identify mild cognitive impairment for Chinese old adults [23,24].

**Table 1:** Demographic characteristics (*N* = 130).

Variables	Value
Age (years)	
Mean±SD	80.12±6.12
Less than 80	63(48.5%)
More than 80	67 (51.5%)
Gender, male (%)	31 (23.8%)
Marital status, married	95 (73.3%)
Height (cm) mean±SD	160.11±8.66
Weight (kg) mean±SD	56.12±11.65
Education (years) mean±SD	4.65±4.96
Disease, thighbone fracture	130 (100%)
Anesthesia, general anesthesia	130 (100%)
ASA class	
Ⅰ	1(0.8%)
Ⅱ	84 (64.6%)
Ⅲ	43(33.1%)
Atropine (mg) mean±SD	0.23±0.34
Solu-Medrol (mg) mean±SD	29.15±27.27
Length of anesthesia (min) mean±SD	130.94±49.81
Length of operation(min) mean±SD	96.53±44.41
Estimated blood loss (mL) mean±SD	319.05±268.72
ADL (points) mean±SD	18.61±6.53
MMSE (points) mean±SD	22.23±5.44

The preoperative MMSE scores in the patients who had postoperative delirium [18.10 ± 8.44, median ± interquartile range (IQR)] were lower than those in the patients who did not have postoperative delirium (24.27 ± 7.87, *P* < 0.001, Mann-Whithney *U* test). A Spearman correlation was used to assess the potential association between preoperative age, ADL scores and MMSE scores, and the severity of delirious symptoms determined by the MDAS. We found that age (*R* = 0.392, *P* = 0.001), preoperative scores of ADL (*R* = 0.476, *P* < 0.001), and preoperative MMSE scores (*R* = -0.777, *P* < 0.001), were all associated with postoperative MDAS scores. Preoperative MMSE (*R* = -0.664, *P* < 0.001) and preoperative ADL scores (*R* = 0.142, *P* < 0.001) remained correlated with the postoperative MDAS scores after adjustments for age, gender and education.

### Optimal cutoff score of MMSE associated with the postoperative delirium

We employed ROC analysis to determine the optimal cutoff score of MMSE associated with postoperative delirium defined by CAM diagnostic algorithm. We obtained 21.4 as the optimal cutoff score of MMSE associated with the postoperative delirium. There were 54 patients who had preoperative MMSE scores less than or equal to 21.4. Twenty-six of these patients (48.1%) developed the postoperative delirium. In another 76 patients, who had MMSE scores greater than 21.4, only eight (10.5%) of them developed postoperative delirium. The AUC of the MMSE prediction model was 0.781 (95%CI: 0.696–0.867, *P* < 0.001). This cutoff score of MMSE (21.4) led to a sensitivity of 76.5% and a specificity of 70.8% for the association with postoperative delirium (Fig 2A).

### Optimal cutoff scores of MMSE associated with postoperative delirium in young-old and old-old participants

Then, we used a ROC analysis to determine the values of the optimal cutoff scores of MMSE associated with postoperative delirium in two age groups: the young-old participants (≤ 80 years old, 75.46 ± 4.69 years, 27.0% male, n = 63) and the old-old participants (> 80 years old, 84.51 ± 3.46 years, 20.9% male, n = 67). There was no difference in education levels between old-old (4.64 ± 4.75 year) and young-old (4.65 ± 5.21 year, *P* = 0.992, student *t*-test) participants. The optimal cutoff score of MMSE associated with postoperative delirium in the old-old participants was still 21.4. With this cutoff score, a sensitivity of 83.8% and a specificity of 62.8% for the association with postoperative delirium were obtained. The area under the curve (AUC) was 0.744 (95% CI: 0.623–0.866, *P* < 0.001).

In the young-old participants, however, the optimal cutoff score of MMSE associated with the postoperative delirium was 18.4, which was lower than that (21.4) in the old-old participants. The AUC at this cutoff score (18.4) was 0.805 (95%CI: 0.668–0.941, *P* < 0.001), achieving a sensitivity of 60.0% and a specificity of 92.5%.

We found that the preoperative MMSE score associated with the postoperative delirium in the young-old participants was lower than that in the old-old participants (Fig 2B). These results also suggested that with same MMSE score, the old-old participants could be more vulnerable to develop postoperative delirium than the young-old participants (Fig 2B).

**Fig 3:**
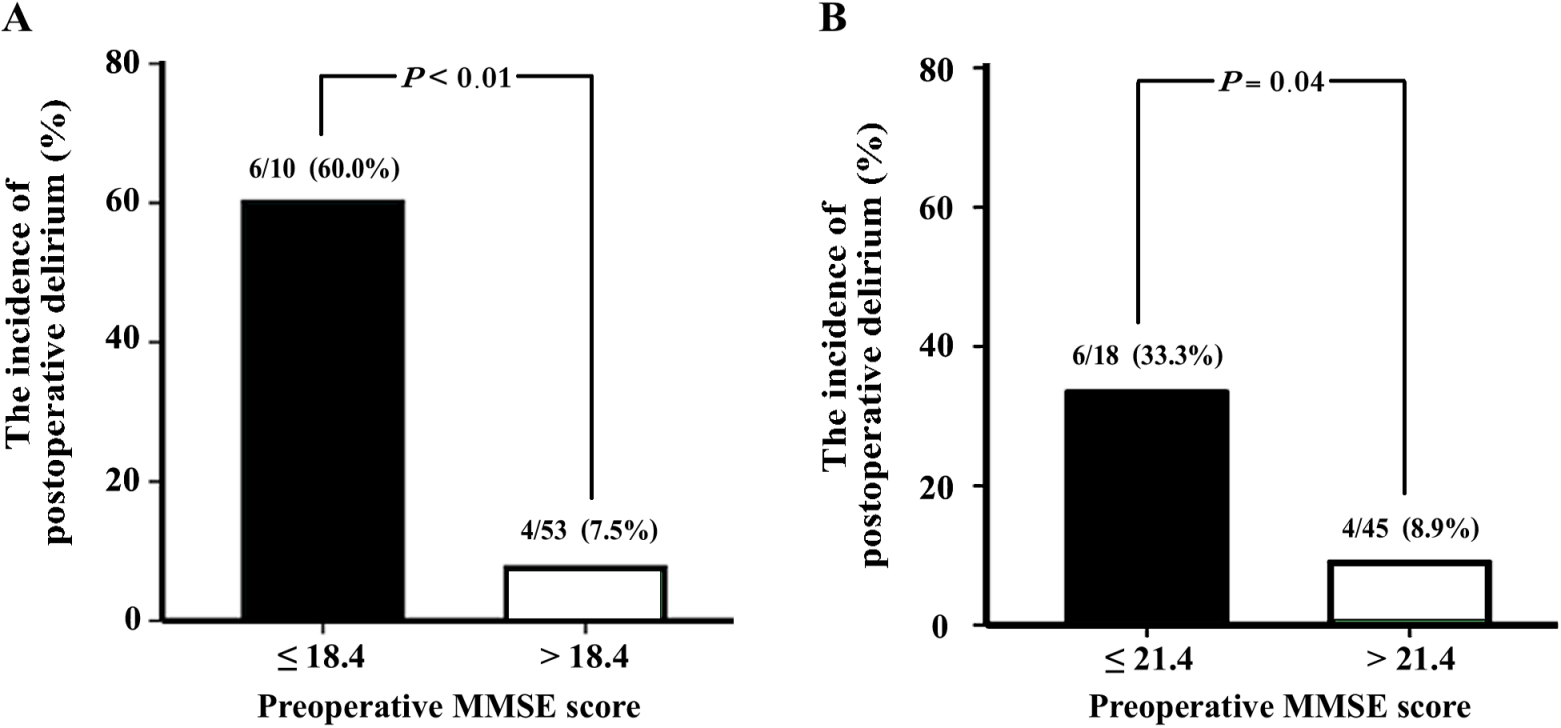
The incidence of postoperative delirium predicted by age-specified cutoff score in young-old participants. (A) Participants who had a MMSE score of less than 18.4 (black bar) have higher incidence of developing postoperative delirium than participants with a MMSE score greater than 18.4 (white bar; *P* < 0.001, *Chi*-square test). (B) The incidence of the postoperative delirium in participants with a MMSE score of less than 21.4 (black bar) is higher than participants who had MMSE scores greater than 21.4 (white bar; *P* = 0.044, *Chi*-square test).

The incidence of postoperative delirium in the patients with MMSE scores less than or equal to 18.4 was 60.0% (6 of 10), whereas the incidence of postoperative delirium in the patients with MMSE scores greater than 18.4 was 7.5% (4 of 53) (Fig 3A). However, when 21.4 MMSE cutoff score was used, the incidence of postoperative delirium in the patients with MMSE scores less than or equal to 21.4 was 33.3% (6 of 18), whereas the incidence of postoperative delirium for the patients with MMSE scores greater than 21.4 was 8.9% (4 of 45) (Fig 3B). Using 18.4 as the cutoff score of the MMSE, the sensitivity remained 60.0%, while the specificity was improved from 77.4% to 92.5%. Thus, these results also suggested that employment of an age-specified MMSE cutoff score might improve the predictive specificity in identifying individuals at high risk of developing postoperative delirium.

## Discussion

We showed that the incidence of the postoperative delirium was 26.2% (34/130 participants) in our current studies, with 44.1% hyperactive delirium (15/34), 38.2% hypoactive delirium (13/34) and 17.7% mixed delirium (6/34). These findings are consistent with the results from previous studies [25,26]. Interestingly, Robinson *et al*. reported that the incidence of postoperative delirium was 43% in their studies, with 1% hyperactive delirium, 68% hypoactive delirium and 31% mixed delirium [27]. The reason for the difference in the incidence and distribution of delirium between the studies by Robinson and our studies is unknown at the present time. The potential explanation for such difference includes age (64 ± 8 years old versus 80 ± 6 years old), gender (97% male versus 23.8% male), types of surgery [multiple surgeries (e.g., cardiac, thoracic, vascular and abdominal surgeries) versus hip surgery] and culture (e.g., the patients in our hospital usually have the accompany by family members). Nevertheless, the incidence of the postoperative delirium in our current studies was consistent with that obtained from the other patients who also had hip surgery [25]. Moreover, Gao *et al*. reported 57.7%, 30.8% and 11.5% of hyperactive, hypoactive and mixed delirium in hip surgery [26].

We found that preoperative risk factors, including age, lower functional state (determined by ADL score), and poorer cognitive function (determined by MMSE), were associated with the severity of postoperative delirium in geriatric patients who had hip surgery under general anesthesia. These results are consistent with findings from previous studies, which also demonstrated that preoperative cognitive state was negatively associated with incidence of postoperative delirium [1,9]. In addition, Guenther *et al*. [28] reported that the combination of age, *Charlson's* co-morbidity index, MMSE scores, and the length of cardiopulmonary bypass might all be used for the prediction of postoperative delirium in patients undergoing cardiac surgery.

In the current studies, we also determined whether different MMSE scores could be associated with postoperative delirium in young-old and old-old adult. We found that the optimal cutoff score of MMSE was age-dependent for the association of the development of postoperative delirium. Specifically, the optimal cutoff score of MMSE in the young-old participants (18.4) was lower than both the general cutoff score of MMSE (21.4) and the cutoff score in the old-old participants (21.4) (Fig 2B). These results suggest that young-old participants might need to have more severe cognitive impairment (MMSE score of 18.4) than the old-old participants (MMSE score of 21.4) in order to develop postoperative delirium after hip surgery under general anesthesia. This difference could be due to the fact that the young-old participants might have less neurodegeneration, higher resistance to neurotoxicity, potentially caused by perioperative factors (e.g., anesthetic isoflurane) [29–31], and a greater brain reserve, as compared to the old-old participants. These findings also suggest that older patients may develop postoperative delirium even with higher cognitive function scores as compared to younger patients. We therefore have postulated that the impaired preoperative cognitive function may be the primary risk factor for the younger patient to develop the postoperative delirium, whereas in older patients, other risk factors have become important as well for the development of postoperative delirium. Future studies to test this hypothesis are warranted. Furthermore, with the employment of this age-specified MMSE cutoff score, the specificity in predicting the incidence of postoperative delirium was improved from 77.4% to 92.5% in the young-old participants. These data showed that the age-specified preoperative MMSE score could better differentiate the individuals at higher risk of developing postoperative delirium than the unified cutoff score of MMSE.

Clinically, these data would suggest that old-old patients (older than 80 years old) might still have high risk to develop postoperative delirium even with minor preoperative cognitive impairment, demonstrated by better MMSE score. Moreover, old-old patients who have pre-operative MMSE score of 21 are likely to develop the postoperative delirium, whereas young-old patient who have pre-operative MMSE score of 18 are likely to develop the postoperative delirium after hip repair surgery.

The studies had several limitations. First, patients were assessed for delirium only up to four days after surgery. This is mainly because delirium usually occurs within two to three days after surgery, and declines rapidly thereafter [1,32]. Regardless, postoperative delirium could have occurred at a later time and the true incidence of delirium could therefore be underestimated. Second, pain and pain medicines may affect the evaluation of MMSE, however, all of the patients received similar pain treatments before the surgery. Finally, we did not assess the effects of the influence of anesthetics on the risk of developing delirium in the current studies. However, the main objective of the current study was to determine whether the different MMSE scores could be associated with postoperative delirium in young-old and old-old adults.

In conclusion, our studies demonstrated the association between preoperative MMSE scores and postoperative delirium (both incidence and severity) in geriatric patients who had repairs of hip fractures under general anesthesia. More importantly, we found that the optimal cutoff score of MMSE associated with the postoperative delirium was 18.4 for the young-old participants (equal to or younger than 80 years-old) and 21.4 for the old-old participants (older than 80 years-old). These findings suggest that the association between preoperative cognitive function and postoperative delirium is age-dependent, and older patients could still develop postoperative delirium even with lesser preoperative cognitive impairment as compared to younger patients, pending further investigation. These findings suggest that the utilization of the age-specific optimal cutoff score of MMSE in predicting postoperative delirium could improve the predictive specificity for the development of postoperative delirium, pending on findings from other confirmation studies. Nevertheless, the findings obtained from these studies would promote more research, which could finally lead to better prevention and intervention of postoperative delirium, especially in geriatric patients.

## Supporting Information

S1 Dataset The necessary database related to original scores of CAM, MMSE and MDAS. (PDF)

## Abbreviations

ADL: Activities of Daily Living
MMSE: Mini-Mental State Examination
CAM: confusion assessment method
ASA: American Society of Anesthesiologists
SD: standard deviation
ROC: receiver operating characteristic
CI: confidence interval
