# Supplementary material for: Different MMSE Score Is Associated with Postoperative Delirium in Young-Old and Old-Old Adults
Source: PLoS One. 2015 Oct 13;10(10):e0139879. doi: 10.1371/journal.pone.0139879 (PMC4603675; doi:10.1371/journal.pone.0139879)
Supplement: S1 Dataset — (PDF) [file pone.0139879.s001.pdf]

Study ID	Age	CAM diagnosis	Preoperative MMSE	MDAS Day1	MDAS Day2	MDAS Day4
**1**	78	0	25	4	2	2
**2**	75	0	24	4	4	2
**3**	79	0	24	1	0	0
**4**	83	0	15	7	6	6
**5**	74	0	28	1	1	1
**6**	69	0	29	1	3	0
**7**	70	0	19	6	3	3
**8**	80	0	14	5	4	6
**9**	80	1	18	8	9	9
**10**	65	0	25	0	0	0
**11**	83	0	21	4	1	1
**12**	93	1	21	11	10	5
**13**	84	1	21	11	2	1
**14**	89	0	17	6	5	3
**15**	90	1	14	13	15	9
**16**	78	0	21	5	1	0
**17**	83	1	16	12	12	5
**18**	86	0	17	5	4	4
**19**	84	0	16	8	6	5
**20**	83	1	11	16	13	13
**21**	80	1	18	19	16	15
**22**	81	0	22	5	5	5
**23**	83	0	23	3	2	2
**24**	91	1	21	14	4	4
**25**	89	0	23	4	4	4
**26**	81	0	18	5	5	5
**27**	83	1	21	18	9	14
**28**	81	0	30	1	0	0
**29**	82	0	21	4	4	4
**30**	63	0	26	1	1	0
**31**	82	0	14	7	6	6
**32**	90	0	23	3	2	2
**33**	81	0	30	1	1	0
**34**	79	0	30	2	1	1
**35**	77	0	25	2	1	1
**36**	90	0	23	6	6	5
**37**	82	1	17	9	5	5
**38**	85	1	8	24	19	20
**39**	79	0	22	5	4	4
**40**	75	0	27	2	2	2
**41**	90	0	13	7	7	6
**42**	67	0	24	2	2	1
**43**	85	0	28	3	3	3
**44**	78	0	29	2	2	1
**45**	70	0	20	6	4	3
**46**	83	0	22	3	2	3
**47**	78	0	25	4	3	3
**48**	80	0	19	5	4	4

**49**	83	0	24	3	2	2
**50**	84	0	24	4	3	3
**51**	77	1	26	9	8	6
**52**	76	0	26	4	2	2
**53**	85	0	23	2	3	3
**54**	79	0	19	6	5	4
**55**	75	0	28	2	2	2
**56**	84	0	28	2	3	3
**57**	75	0	25	1	1	1
**58**	88	1	27	11	5	2
**59**	81	0	19	3	3	2
**60**	72	0	26	4	3	3
**61**	80	0	22	4	3	3
**62**	81	1	9	17	17	18
**63**	82	1	13	6	17	13
**64**	76	0	29	1	1	1
**65**	70	0	27	5	5	2
**66**	77	0	22	3	4	2
**67**	73	0	27	1	1	1
**68**	81	0	22	4	5	5
**69**	79	0	29	2	2	1
**70**	77	0	29	0	0	0
**71**	83	1	15	15	11	4
**72**	85	0	19	5	5	6
**73**	82	0	15	7	6	6
**74**	84	1	18	2	11	4
**75**	82	0	23	6	7	5
**76**	88	1	13	8	15	9
**77**	80	0	25	4	4	4
**78**	81	1	20	15	19	17
**79**	70	0	29	2	2	2
**80**	78	0	19	5	5	5
**81**	68	0	30	1	1	0
**82**	78	0	30	1	4	1
**83**	84	0	27	2	2	1
**84**	80	0	30	1	1	1
**85**	81	1	18	10	4	3
**86**	90	0	29	1	2	0
**87**	79	1	12	13	15	8
**88**	81	0	25	2	2	2
**89**	81	0	28	1	2	2
**90**	80	0	22	3	1	1
**91**	85	0	20	5	7	6
**92**	82	0	18	8	5	4
**93**	65	0	25	7	5	5
**94**	70	1	24	7	27	15
**95**	79	0	16	5	4	4
**96**	78	0	20	5	5	4

**97**	82	0	25	3	1	1
**98**	86	1	16	12	3	4
**99**	76	1	24	5	4	14
**100**	78	1	13	17	14	15
**101**	81	0	22	5	10	7
**102**	80	0	10	9	10	11
**103**	75	0	29	0	0	1
**104**	85	0	27	1	1	1
**105**	79	0	21	2	4	2
**106**	68	0	29	0	1	0
**107**	82	1	21	16	22	25
**108**	73	0	30	3	1	1
**109**	81	0	28	2	4	0
**110**	79	0	29	3	2	1
**111**	84	0	19	5	5	1
**112**	77	0	25	4	4	4
**113**	77	1	18	21	17	6
**114**	90	0	28	1	1	1
**115**	81	0	26	3	3	3
**116**	78	0	29	3	1	1
**117**	82	0	26	3	3	2
**118**	66	0	30	1	0	0
**119**	85	1	24	13	1	12
**120**	93	1	23	7	7	12
**121**	79	0	27	2	3	2
**122**	86	0	19	6	6	7
**123**	84	1	26	13	3	7
**124**	77	1	25	3	1	1
**125**	80	1	12	17	12	13
**126**	85	1	20	5	9	14
**127**	94	1	12	9	13	13
**128**	86	0	24	4	0	0
**129**	67	0	29	2	2	2
**130**	80	0	16	9	4	6
